# Application value of Early-Follicular Phase Long-Acting Gonadotropin-Releasing Hormone Agonist Long Protocol in patients with resistant ovary syndrome

**DOI:** 10.1186/s12884-023-05477-w

**Published:** 2023-03-15

**Authors:** Fan Zhang, Huixia Zhang, Hui Du, Xin Li, Haixia Jin, Gang Li

**Affiliations:** Centre for Reproductive Medicine, Henan Province Key Laboratory of Reproduction and Genetics, The First Affiliated Hospital of Zhengzhou University, Zhengzhou, 450052 Henan China

**Keywords:** Resistant ovary syndrome, Early-follicular short-acting GnRH agonist long protocol (EFLL), Controlled ovarian stimulation, Oocyte retrieval

## Abstract

**Background:**

Resistant ovarian syndrome(ROS) is a rare disease. It is difficult to diagnose and treat. Most of the literature reports on assisted pregnancy treatment for ROS patients are individual case reports. In this paper, the ovulation stimulation protocol and assisted pregnancy process of ROS infertile patients in our reproductive center were summarized and analyzed to provide information and support for the clinical treatment of ROS patients.

**Methods:**

From January 2017 to March 2022, assisted reproductive technology treatments and clinical characteristics parameters of six patients with ROS were retrospectively reviewed. Based on controlled ovarian stimulation protocols, these stimulation cycles were separated into four groups: Early-Follicular Phase Long-Acting Gonadotropin-Releasing Hormone Agonist Long Protocol (EFLL) group (*n* = 6), Progestin Primed Ovarian Stimulation(PPOS) protocol group (*n* = 5), mild-stimulation protocol group (*n* = 2), and Natural cycle protocol group (*n* = 3).

**Results:**

A total of 16 cycles of ovulation stimulation were carried out in 6 patients with ROS. A total of 19 oocytes were retrieved, as well as 13 MII oocytes, 11 two pronuclear(2PN) fertilized embryos, and 8 excellent embryos. The oocytes acquisition rate was 50% and the fertilization rate of 2PN was 57.9%, and the excellent embryo rate was 72.7%. The EFLL protocol obtained 17 oocytes, 12 MII oocytes, 11 2PN fertilized embryos, and 8 excellent embryos; the mild-stimulation protocol obtained 1 oocyte; the Natural cycle protocol obtained 1 oocyte, and oocytes were not matured after in vitro maturation (IVM); the PPOS protocol obtained no oocytes. Compared with three other protocols, The fertilization rate of 2PN (64.7%) and excellent embryo rate (72.7%) in the EFLL protocol were higher than those of other protocols(0%). Two fresh cycle embryo transfers resulted in live births, while two frozen-thawed embryo transfer cycles resulted in one live birth and one clinical pregnancy using the EFLL protocol.

**Conclusion:**

Although the current study is based on a small sample of participants, the findings suggest that the EFLL protocol can be employed for ovarian stimulation and may result in a live birth in ROS patients.

## Background

Resistant ovarian syndrome(ROS), also known as insensitive ovary syndrome or savage syndrome, is a rare disease that has been initially reported in 1967 by Jones and Ruehsen [1]. Patients with high gonadotropin (Gn) and low estrogen levels but normal ovarian reserves, such as normal anti-Mullerian hormone (AMH) and inhibin B levels, often exhibit infertility. Transvaginal ultrasound reveals normal antral follicles [2]. The chromosome karyotype examination of the patients was generally normal [3]. Clinically, patients with resistant ovary syndrome should be distinguished from those with premature ovarian failure (premature ovarian insufficiency, POI) [4]. The latter refers to ovarian function loss before the age of 40, followed by clinical symptoms of menstruation problems and elevated gonadotropin FSH > 25 IU/L (interval > 4 weeks), and reduced estrogen. The number of antral follicle count (AFC) and the serum AMH levels have decreased to a level where they are undetectable or low. Patients' ovarian follicles respond poorly or not enough to endogenous or exogenous Gn. Infertility therapy in these people is sometimes tortuous and difficult. With the advancement of assisted reproductive technology, the use of different ovulation stimulation protocols [5] and oocyte in vitro maturation (IVM) technologies [6] in patients with resistant ovary syndrome has been reported. At present, the majority of the reported ovulation stimulation protocols use mild-stimulation, Natural cycle, super-long pituitary down-regulation, and other protocols [3, 7, 8]. Nevertheless, there is still a considerable amount of uncertainty associated with the relationship between ROS and ovulation stimulation protocols.

The early follicular long-acting GnRH-a long protocol (EFLL) is frequently utilized since it has been linked to increased endometrial receptivity, implantation, and clinical pregnancy rates [9–11]. The size and number of the antral follicles were monitored from early in the menstrual cycle. A single intramuscular injection of Gonadotropin-releasing hormone agonists was given when the diameter of the antral follicles was < 5 mm. After 28 days (no ovarian cysts > 8 mm; E2 > 50 pg/L) before commencing hormone stimulation, the downregulation was confirmed. During the stimulation, no further GnRH analogues were administered. According to Ron-El et al. [12], the EFLL group had greater metaphase II (MII) oocyte rates (76% vs. 68%) and more embryos with excellent morphology (69% vs. 62%) than the classical luteal phase long procedure group. This also accords with our earlier observations [13]. Data from 5197 IVF/ICSI cycles performed at our center from May 2015 to 2016 showed that the follicular phase long-acting GnRH-a long protocol was significant compared to the classical luteal phase long protocol in terms of the number of oocytes obtained (12.93 ± 7.51 vs. 12.87 ± 7.45), the rate of fertilization (45.34% vs. 37.68%) and the clinical pregnancy rate (63.72% vs. 52.67%) [13].

This paper will go through the various ovulation stimulation protocols used in our center for ROS patients. We present evidence of the feasibility of using the long-acting long protocol for ovulation stimulation during the follicular phase in patients with ROS, aiming to provide reference and support for the clinical treatment of such patients.

## Methods

### Patients

The research data in this paper is drawn from the Clinical Reproductive Medicine Management System/Electronic Medical Record Cohort Database (CCRM/EMRCD) in the Reproductive Medical Center, the First Affiliated Hospital of Zhengzhou University. Six patients with ROS were diagnosed in the outpatient clinic of the reproductive center of our hospital from January 2017 to March 2022. It was observed that all six patients included in the study had the following characteristics: age < 40 years, basal FSH > 10 U/L on multiple tests, antral follicle count > 7 on ultrasound, and serum antimullerian hormone (AMH) > 1.1 ng/L. The peripheral blood karyotype was normal. Hysteroscopy showed normal morphology of the uterine cavity. The spouse's semen routine examination did not show any abnormality. Routine semen testing was performed according to the WHO Laboratory Manual for the Examination and Processing of Human Semen (6th Edition) criteria [14].

### Controlled ovarian stimulation(COS) protocols

The appropriate ovulation induction protocol was selected based on a comprehensive assessment of the patient's age, body mass index (BMI), basal sex hormone levels, ovarian reserve, compliance, and previous medical history. There is no specific preference regarding the first ovulation induction protocol. Following the patient's wishes and the therapeutic effect, another ovulation induction protocol was chosen when the previously selected protocol failed to produce satisfactory results.

EFLL Protocol: The standard full dose (3.75 mg) of Long-acting GnRH-a (Diphereline, Ipsen, France) was given on days 2–4 of menstruation. Patients were monitored by transvaginal sonography after 28 days and serum sex hormone levels were measured. Gn was used for controlled ovarian stimulation once the downregulation criteria were satisfied (no functional cyst, follicle size 3–5 mm under ultrasound, estradiol (E2) < 30 µg/mL, luteinizing hormone (LH) < 5 IU/L).

Progestin Primed Ovarian Stimulation(PPOS) Protocol: Oral progesterone (MPA, Zhejiang Xianju Pharmaceutical Co, Ltd.) 10 mg/d from the days 2–4 of menstruation until the trigger day, combined with urinary human menopausal gonadotropin (hMG, Shanghai Lizhu Pharmaceutical Co, Ltd.) 75-150 IU, combined with serum estradiol. At the appropriate time, the dosage was modified depending on serum estradiol levels and follicle monitoring results. Alternatively, depending on the diameter of small follicles (< 8-10 mm), hMG 150–300 IU is added for initiation, including the endocrine and follicle monitoring results, a few days after the oocyte retrieval process.

Mild-stimulation protocol: There is no need for patients to take gonadotropin daily as part of this protocol. Starting on day 2–3 of the menstrual cycle, take 2.5–5 mg/d letrozole (Jiangsu Hengrui Pharmaceutical Co., Ltd., China) with an additional hMG 75-150 IU depending on follicle growth.

Natural cycle protocol: depending on the duration of the menstrual cycle, vaginal ultrasound monitoring can start on days 6–8 of the menstrual cycle, with an eye on LH, E2, and P (particularly E2) during the monitoring phase. Once the E2 value failed to rise and showed a decreasing trend, 10,000 IU of HCG was administered, and oocyte retrieval procedures were performed 36 h later.

### Oocyte retrieval and In vitro fertilization procedures

Oocytes were collected and cultivated in vitro 36 to 37 h after u-hCG (Livzon, Guangzhou, China) injection or without hCG injection depending on follicular growth, using vaginal ultrasound guidance.

Immature oocytes (including metaphase I and germinal vesicle stages) were observed under a mild scope and cultured in IVM culture media (SageMedia TM Quinn's series reagents, USA) for 24 h at 37 °C and 6% CO2. Mature oocytes (metaphase II)with obvious first polar body expulsion were subjected to ICSI, and the presence of 2PN and 2 polar bodies in the cytoplasm was observed at 16-20 h as normal fertilization, followed by observation of oogenesis and oogenesis embryo morphology, and embryo assessment was calculated using the Peter scoring system criteria [6]. Embryos with a score of I or II were deemed excellent.

### Embryo transfer and clinical pregnancy examination

Fresh cycle transfer or frozen embryos were selected based on hormone levels and endometrial thickness, and luteal phase support, such as oral Daphton (AbbottBiologicalsB.V, Holland) and vaginal progesterone gel (Merck Sherano, Switzerland), was administered following transfer. On the 14th day following transplantation, serum HCG was detected, and on the 35th day, a B-ultrasound examination was performed. The standard of clinical pregnancy is an observation of the intrauterine gestational sac and the fetal heartbeat [15].

## Results

### Basic clinical characteristics of patients with ROS

The mean age of the 6 patients with ROS was (29.67 ± 4.32) years; years of infertility (3.97 ± 3.20), as well as a BMI range of 17.55–24.1 kg/m^2^. Basal AFC (13 ± 4.34); basal FSH (28.14 ± 10.20); AMH (2.75 ± 1.53) ng/ml. There were four cases of primary infertility and two cases of secondary infertility. Table 1 shows the specifics.

**Table 1:** Basic clinical characteristics of patients with ROS

Case	Age	Type of infertility	Years of infertility	BMI (kg/m2)	Basal AFC	Basal FSH (IU/l)	AMH (ng/ml)
1	28	Primary	2	19.6	7	25.78	1.73
2	28	Secondary	4	19.5	16	25.35	2.52
3	30	Secondary	0.83	22.7	12	46.95	1.46
4	24	Primary	4	17.55	17	20.32	5.71
5	37	Primary	10	23.4	9	19.11	2.84
6	31	Primary	3	24.1	17	31.3	2.28

*BMI* Body mass index, *AFC* Antral follicle count, *AMH* Anti-Müllerian hormone, *NA*: Not applicable

### Ovulation stimulation cycles and outcomes in patients with ROS

Table 2 shows that the patient went through a total of 16 ovulation cycles. Three cycles were canceled due to insufficient follicular growth. An HCG trigger was used in all oocyte retrieval cycles, and fertilization was done via ICSI. Nineteen oocytes were retrieved, 13 of which were mature, 11 of which were fertilized with 2PN, and 8 excellent quality embryos were produced. Using the EFLL protocol six times yielded a total of 17 oocytes and 12 mature oocytes. two pronuclear fertilized 11 oocytes, resulting in the formation of eight high-quality embryos.1 oocyte was obtained in 1 mild stimulation cycle with poor quality oocytes that did not fertilize. 1 oocyte was obtained in 1 Natural cycle that did not mature after IVM culture. Two fresh cycle embryo transfers using the EFLL protocol resulted in live births.

**Table 2:** Ovulation stimulation cycles and outcomes in patients with ROS

case	cycle	Stimulation protocol	Days of Gn stimulation	Gn total dose (IU)	ovulation trigger(IU)	Serum E2 on trigger day (pg/ml)	No. of retrieved oocytes	No. of MII oocytes	The way of fertilization	No. of fertilization	No. of excellent embryo	Result
1	1	EFLL	8	2400	u-HCG10000	109.1	2	2	ICSI	2	2	Live born after fresh double embryo transfer
2	2	mild-stimulation	15	4500	u-HCG10000	95.79	1	1	ICSI	0	0	No fertilization
	3	EFLL	11	3300	u-HCG10000	43.36	3	0	ICSI	2	1	Three MI stage oocytes were obtained, 2 oocytes fertilized after IVM, and finally 2 embryo cryopreserved
	4	Natural cycles	0	0	u-HCG10000	142.8	0	NA	NA	0	0	No oocytes were obtained
3	5	PPOS	15	5700	NA	NA	NA	NA	NA	NA	NA	No follicle growth, cancelled cycle
	6	EFLL	20	5725	u-HCG10000	77.71	1	1	ICSI	1	1	One embryo cryopreserved
	7	PPOS	8	2400	u-HCG10000	69.33	0	NA	NA	0	0	No oocytes were obtained
	8	Natural cycles	0	0	u-HCG10000	99.06	0	NA	NA	0	0	No oocytes were obtained
	9	PPOS	1	300	u-HCG10000	93.59	0	NA	NA	0	0	No oocytes were obtained
	10	EFLL	27	12,026	u-HCG10000	126.9	4	4	ICSI	2	2	Two embryo cryopreserved
4	11	EFLL	19	5200	u-HCG10000	219.8	3	1	ICSI	0	0	One MII oocyte was obtained and did not cleavage after ICSI. Two MI oocytes were obtained and immature after IVM
	12	PPOS	7	1800	NA	NA	NA	NA	NA	NA	NA	No follicle growth, cancelled cycle
	13	Natural cycles	0	0	u-HCG10000	256.1	1	0	NA	0	0	One MI oocyte was obtained and immature after IVM culture
	14	mild-stimulation	7	1200	u-HCG10000	323	0	NA	NA	0	0	No oocytes were obtained
5	15	EFLL	21	5700	250ug r-hCG + 2000 u-HCG	362.7	4	4	ICSI	4	2	Live born after fresh double embryo transfer
6	16	PPOS	38	6750	NA	NA	NA	NA	NA	NA	NA	No follicle growth, cancelled cycle

*EFLL* The early follicular-phase long-acting GnRH-agonist long protocol, *Gn* Gonadotropin, *IU* International units, *E2* Estradiol, *ICSI* Intracytoplasmic sperm injection, *IVM* In vitro maturation, *u-hCG* Urinary HCG, *HMG* Human menopausal gonadotropin, *MII* Mature oocytes, *MI* MetaphaseIoocytes, *NA* Not applicable

### Comparison of the results of different ovulation stimulation protocols

As shown in Table 3, the follicular phase long-acting treatment was performed in six of the sixteen ovulation cycles, whereas other protocols were used in ten. The total Gn of the EFLL protocol was 5450 IU (3075, 7300) greater than that of other protocols 2400 IU (1200, 5700), and the HCG daily estrogen level was 118 pg/ml (69.12, 255.52) slightly higher than that of other protocols 99.06 pg/ml (93.59, 256.1). The EFLL protocol obtained seventeen oocytes, with 12 mature oocytes, a 2PN fertilization rate of 64.7%, and an excellent embryo rate of 72.7%, while the other protocols obtained just two oocytes. The most striking observation from the data comparison was that none of the retrieved oocytes completed fertilization and transferred embryos in the other protocols. Patients using the EFLL protocol for ovulation stimulation achieved live births in two fresh transfer cycles, followed by two frozen-thawed embryo transfer cycles, one live birth, and one clinical pregnancy.

**Table 3:** Comparison of the results of different ovulation stimulation protocols

Stimulation protocol	No. of cycle	Gn total dose (IU)	Serum E2 on trigger day (pg/ml)	No. of retrieved oocytes	No. of MII oocytes	2PN Fertilization rate %(n/n)	excellent embryo rate %(n/n)	No.of clinical pregnancy	No.of live birth
EFLL protocol	6	5450 (3075, 7300) *	118 (69.12, 255.52) *	17	12	64.7 (11/17)	72.7 (8/11)	4	3
Other protocol	10	2400 (1200, 5700) *	99.06 (93.59, 256.1) *	2	1	0 (0/0)	0 (0/0)	0	0
Total	16	2850 (525, 5700) *	109.10 (85.65, 237.95) *	19	13	57.9 (11/19)	72.7 (8/11)	4	3

^*^Median (Lower quartile,Upper quartile), EFLL: The early follicular-phase long-acting GnRH-agonist long protocol, 2PN Fertilization rate = No.of 2PN fertilization / No. of retrieved oocytes × 100%, Excellent embryo rate = No.of embryos with a score of I or II / No.of cleavage embryos × 100%

## Discussion

### Advances in the pathogenesis and treatment of ROS

Patients with ROS have a normal ovarian reserve, but their follicles do not react to endogenous gonadotropins. There is generally no follicular growth and no corresponding increase in estradiol levels, resulting in negative feedback to the pituitary gland and elevated gonadotropins. The relative role of etiology in the literature on ROS has been hotly debated. In 2019, two novel pathogenic FSH receptor (FSHR) variants causing resistant ovary syndrome were reported [16], although other studies [17–19] showed that gonadotropin FSH receptor gene expression is normal in patients with ROS. Granulosa cells were confirmed to be normal. It is most likely that patients' poor response to reproductive therapy does not depend on their response pattern and FSHR variations in granulosa cells [17, 20]. With the constant advancement of assisted reproductive technologies, the treatment of infertility in patients with resistant ovary syndrome has been reported several times in recent years. In recent years, the oocyte in vitro maturation (IVM) technique has been reported several times [8, 21, 22] in the infertility treatment of patients with ROS. Our findings are in agreement with Nikolay’s studies, which demonstrated pregnancy and live birth in a 23-year-old patient with ROS following IVM and PGT-A treatment [23]. In case 2, three metaphase I oocytes were retrieved, two of which were fertilized following IVM and eventually formed two excellent embryos, followed by freeze–thaw embryo transfer, and the patient had a live birth. These findings provide support to the hypothesis that oocytes generated using the IVM technique in ROS patients are capable of meiosis and fertilization. IVM technique can result in euploidy embryos, excellent embryos, and live birth in patients suffering from ROS. However, we observed that immature oocytes retrieved from two ovulation stimulation cycles remained immature during IVM culture. This disparity might be explained by the low quality of oocytes retrieved in this cycle. A study reported [24] that laparoscopic ovariectomy was used to treat patients with ROS, and the Hippo signaling system was thought to suppress follicular growth. The ovarian cortex was incised to impair Hippo signaling, which is responsible for follicle growth [25]. This might be a therapy option for those suffering from ROS.

### Exploration of the value of applying EFLL protocol in patients with ROS

Patients with elevated basal FSH have poorer overall ovarian responsiveness and decreased oocyte counts [26, 27]. Ovulation stimulation protocols are usually chosen from Natural cycle protocols, mild-stimulation protocols, and PPOS [28] to avoid excessive pituitary suppression and decreased ovarian sensitivity to Gn, which can result in inferior outcomes following assisted conception. Letrozole coupled with HMG has been reported [7] to be utilized in the treatment of infertility in patients with ROS.

Patients demonstrated dominant follicular growth and ovulation, leading to full-term pregnancy and successful delivery. This contrasts with our findings, in which we used letrozole in combination with HMG to stimulate ovulation in ROS patients before in vitro fertilization-embryo transfer therapy, and all patients had inadequate follicular development and failed to assist in conception. ROS patients had poor ovarian sensitivity to endogenous Gn and high baseline FSH levels. Our center attempts to use the above-mentioned ovulation stimulation protocol resulted in a limited number of oocytes and no transferrable embryo formation. Long-acting GnRH-a protocol, one of the commonly used controlled ovulation stimulation protocols in vitro fertilization-embryo transfer technique for fertility treatment, is commonly applied in patients with combined endometriosis, adenomyosis, and uterine fibroids because it increases embryo implantation rate and significantly improves clinical pregnancy rate [29]. Previous research at our center has demonstrated that the follicular phase long-acting GnRH-a long protocol is comparable to the classical luteal phase long protocol in terms of the number of oocytes obtained(12.93 ± 7.51 vs. 12.87 ± 7.45), with significantly higher rates of fertilization(45.34% vs. 37.68%) and clinical pregnancy(63.72% vs 52.67%) than the classical luteal phase long protocol [13]. There was no significant difference in clinical pregnancy and live birth rates between patients with an unanticipated low response and patients with expected low response aged < 35 years using the long follicular phase protocol, which is a more desirable option for young patients with low ovarian response [30]. In this study, We compared the results of ovulation stimulation in ROS patients utilizing the follicular phase long-acting protocol to other protocols. We discovered that ROS patients who used the EFLL protocol could obtain oocytes that would develop into excellent embryos following ICSI and eventually result in live births.

In 2014 Xu [5] reported a case of ROS infertility. Her treatment approach started with 2 Natural cycles of oocyte retrieval, however, both follicles were expelled prematurely. The following luteal phase-long technique was utilized to obtain high-quality embryos, but no pregnancy resulted. Finally, the follicular phase-long protocol was chosen, and two embryos were transferred, with ultrasound indicating a double pregnancy 30 days later. In one of the patients with ROS reported by Yang Rui et al. [3] in 2020, ovulation stimulation was used with letrozole + hMG without pregnancy. Later, she was converted to the EFLL protocol with Gn15d. 16 oocytes and 9 embryos were retrieved. Following the transfer of one 4BC blastocyst from the frozen-thawed embryo, live birth was achieved. The results of our study are consistent with those of previous studies. A single injection of long-acting GnRH-a 3.75 mg during the follicular phase could effectively inhibit the secretion of endogenous FSH and LH in ROS patients. Satisfactory pituitary downregulation could be achieved, with subsequent oocyte retrieval. Down-regulation of the long-acting GnRH-a may have an impact on oocyte development and maturation [31]. A randomized controlled trial found that utilizing a long-acting long protocol in the follicular phase increased the expression of endometrial tolerance markers HOXA10, MEIS1, and LIF, resulting in an increased live birth rate in fresh embryo transfer cycles [11]. We also observed live births in ROS patients with fresh cycles of transfer following two follicular phases of a long-acting protocol. Hence, it is plausible to hypothesize that the EFLL protocol descending pattern may improve endometrial tolerance in ROS patients by prolonging pituitary descending time, which is conducive to embryo implantation and subsequently affects pregnancy outcomes.

### Considerations regarding assisted reproductive therapy for ROS patients

Patients with ROS may be in a stage between the start of reproductive function decrease and follicular arrest. Endocrine and follicular development problems are heterogeneous, with varied periods of onset and rates of decline in reproductive function. Diagnosing and treating is difficult. Adoption of proper pregnancy-assistance strategies is therefore critical for this group of patients.

Limitations to this pilot study need to be noted. In this study, the sample size is small, and due to limitations in objective conditions, only six patients were included from a single institution, so the representativeness of the sample is limited. Our next step will be to collaborate with other institutions to increase the sample size for further verification. Since ROS patients are fluctuating in their sensitivity to Gn and are often resistant, in addition to their different economic circumstances, we implement a variety of ovulation induction protocols to facilitate pregnancy to achieve the best possible outcome.

## Conclusions

This study set out to determine the effect of different protocols of ovulation stimulation on patients with ROS. Even though the current research has a limited sample size, the findings suggest that the EFLL protocol can lead to oocyte acquisition in patients with ROS and may improve endometrial tolerance, leading to good pregnancy outcomes. Prospective research is needed to confirm the findings.

## Data Availability

Data regarding any of the subjects in the study has not been previously published unless specified. Data will be made available to the editors of the journal for review or query upon request. The data underlying this article cannot be shared publicly due to the privacy of individuals that participated in the study. The data will be shared on reasonable request to the corresponding author.

## Abbreviations

ROS: Resistant ovarian syndrome
EFLL: Early-Follicular Phase Long-Acting Gonadotropin-Releasing Hormone Agonist Long Protocol
PPOS: Progestin Primed Ovarian Stimulation) Protocol
AMH: Anti-Mullerian hormone
BMI: Body mass index
AFC: Antral follicle count
Gn: Gonadotropin
E2: Estradiol
FSH: Follicle-stimulating hormone
LH: Luteinizing hormone
IVF: In vitro fertilization
GnRH-a: Gonadotropin releasing hormone analogue
ICSI: Intracytoplasmic sperm injection
IVM: In vitro maturation
HMG: Human menopausal gonadotropin
MII: Mature oocytes
MI: MetaphaseIoocytes
2PN: Two pronuclear
